# Midostaurin Modulates Tumor Microenvironment and Enhances Efficacy of Anti-PD-1 against Colon Cancer

**DOI:** 10.3390/cancers14194847

**Published:** 2022-10-04

**Authors:** Cheng-Ta Lai, Chih-Wen Chi, Shu-Hua Wu, Hui-Ru Shieh, Jiin-Cherng Yen, Yu-Jen Chen

**Affiliations:** 1 Institute of Pharmacology, College of Medicine, National Yang Ming Chiao Tung University, Taipei 112304, Taiwan; 2 Division of Colon and Rectal Surgery, Department of Surgery, MacKay Memorial Hospital, Taipei 104217, Taiwan; 3 Department Medical Research, MacKay Memorial Hospital, New Taipei City 251020, Taiwan; 4 Department of Radiation Oncology, MacKay Memorial Hospital, Taipei 104217, Taiwan; 5 Department of Artificial Intelligence and Medical Application, MacKay Junior College of Medicine, Nursing and Management, Taipei 112021, Taiwan; 6 Department of Medical Research, China Medical University Hospital, Taichung 404332, Taiwan

**Keywords:** colon cancer, midostaurin (PKC412), tumor microenvironment, anti-programmed cell death protein 1 (PD-1), cGAS-STING signaling

## Abstract

**Simple Summary:**

Colon cancer is one of the most common types of cancer worldwide. Immune checkpoint inhibitors have promising effects on various types of cancers with limited efficacy in colon cancer. Midostaurin (PKC412) is currently used for the treatment of patients with acute myeloid leukemia harboring FLT3-mutation. The aim of this study was to assess the potential effect of midostaurin on the modulation of TME and the efficacy of anti-PD-1 against colon cancer. We showed midostaurin inhibited colorectal adenocarcinoma cell growth and induced multinucleation and micronuclei formation. Midostaurin inhibited colorectal adenocarcinoma cell growth associated with the formation of dsDNA and ssDNA; the up-regulation of mRNA expression of cGAS, STING, IRF3, and IFNAR1; the down-regulation of Trex-1, c-Kit, and Flt3 protein expression. The tumor-implanted model displayed a combination of midostaurin-enhanced efficacy of anti-PD-1 to suppress tumor growth. In TME, midostaurin diminished Treg cells and increased M1 macrophage. The expressions of STING and INFβ proteins were elevated in the tumor specimens. Our results suggest that midostaurin may have the potential to enhance immunotherapy in clinical practice.

**Abstract:**

Immunotherapy modulating the tumor microenvironment (TME) immune function has a promising effect on various types of cancers, but it remains as a limited efficacy in colon cancer. Midostaurin (PKC412) has been used in the clinical treatment of fms-like tyrosine kinase 3 (FLT3)-mutant acute myeloid leukemia and has demonstrated immunomodulatory activity. We aimed to evaluate the effect of midostaurin on the modulation of TME and the efficacy of anti-programmed cell death protein 1 (PD-1) against colon cancer. Midostaurin inhibited the growth of murine CT26 and human HCT116 and SW480 cells with multinucleation and micronuclei formation in morphology examination. The cell cycle arrested in the G2/M phase and the formation of the polyploid phase was noted. The formation of cytosolic DNA, including double-strand and single-strand DNA, was increased. Midostaurin increased mRNA expressions of cGAS, IRF3, and IFNAR1 in colorectal adenocarcinoma cells and mouse spleen macrophages. The protein expressions of Trex-1, c-KIT, and Flt3, but not PKCα/β/γ and VEGFR1, were down-regulated in midostaurin-treated colorectal adenocarcinoma cells and macrophages. Trex-1 protein expression was abrogated after FLT3L activation. In vivo, the combination of midostaurin and anti-PD-1 exhibited the greatest growth inhibition on a CT26-implanted tumor without major toxicity. TME analysis demonstrated that midostaurin alone decreased Treg cells and increased neutrophils and inflammatory monocytes. NKG2D^+^ and PD-1 were suppressed and M1 macrophage was increased after combination therapy. When combined with anti-PD-1, STING and INFβ protein expression was elevated in the tumor. The oral administration of midostaurin may have the potential to enhance anti-PD-1 efficacy, accompanied by the modulation of cytosolic DNA-sensing signaling and tumor microenvironment.

## 1. Introduction

Colon cancer is one of the most common cancers and ranks third in incidence and second in mortality of malignancies worldwide [1]. Colon cancer frequently occurs in middle-aged men; good prognosis is usually observed in localized disease [2]. Surgery, chemotherapy [3,4,5], and target therapy [6] remain the main treatment modalities. Unfortunately, 30–40% of patients develop recurrence after surgical resection and 50% of patients with colon cancer appear hepatic with lung metastases, leading to at least two-thirds of death in colon cancer [7].

The tumor microenvironment (TME) is the cellular level of environment in which malignant cells and non-cancerous cells interact in a complicated context [8]. Tumor-infiltrating cells include immune cell lineages, such as T cells, natural killer (NK) cells, myeloid derived suppressor cells (MDSC), macrophages, and dendritic cells (DC), may produce soluble mediators to modulate the anti-tumor immunity in TME [9]. Among T cells, cytotoxic T lymphocytes (CTL) and regulatory T cells (Treg) are distinct subtypes with tumor-inhibitory and immune-suppressive activities, respectively. The tumor-associated macrophages (TAM) can be classified into two subgroups, namely M1 with pro-inflammatory and M2 with anti-inflammatory phenotypes [9].

In the recent decades, the understanding of the cellular and molecular interaction mechanisms between immune and cancer cells has led to developments in immunotherapy [10]. Immune checkpoints are negative regulatory molecules with immunosuppressive effects, which are induced to prevent excess inflammation and to maintain self-tolerance. One of the most investigated immune checkpoint pathways is the programmed cell death protein 1 (PD-1) expressing on T cells that binds its ligands programmed cell death 1 ligand 1 (PD-L1) and PD-L2 expressing on antigen presenting cells to break the immune response [11]. Several immunotherapy drugs developed as immune checkpoint inhibitors (ICIs) have been approved for cancer treatment [12]. Patients with defective microsatellite instability (MSI) due to a DNA mismatch repair (MMR) system with abnormal insertion or deletion of repeating units may have the potential of susceptibility to the immunotherapy [13]. However, the therapeutic efficacy for patients with colon cancer is still undergoing clinical investigations.

The cyclic guanosine monophosphate (GMP)-adenosine monophosphate (AMP) synthase (cGAS)-stimulator of the interferon genes’ (STING) pathway is a DNA-sensing pathway that activates the host innate immune response to produce type I interferon (IFN) and inflammatory cytokines against foreign pathogens [14]. Genotoxic stresses such as chemotherapeutics or irradiation induce DNA damage in the nucleus, resulting in the accumulation of cytosolic DNA as damage-associated molecular pattern (DAMP) signaling. The damaged nucleus may generate single-strand DNA (ssDNA) and double-strand DNA (dsDNA) and transport to cytosol [15]. The cytosolic DNA can bind to cGAS to activate the cGAS-STING-type I IFN signaling in both tumor- and antigen-presenting cells [14,16,17]. However, the cytosolic DNA is degraded by three-prime repair exonuclease 1 (Trex1), which is activated by stresses such as high-dose ionizing radiation to mitigate the cytosolic DNA-induced anti-tumor immunity [18,19].

Midostaurin (*N*-benzoyl staurosporine) is a multiple kinase inhibitor that was first developed to improve the selectivity of staurosporine on protein kinase c (PKC) [20]. During drug development, the other targets of midostaurin were found, including VEGF receptor tyrosine kinase [21], fms-like tyrosine kinase 3 (FLT3)-catalyzed transphosphorylation [22], and KIT proto-oncogene receptor tyrosine kinase (KIT) [23,24]. Based on the results of clinical trials, the FDA approved its indications in FLT3-mutated AML and KIT-mutated systemic mastocytosis patients in 2017 [22,25,26,27,28].

In the present study, we aimed to evaluate the effects and putative mechanisms of midostaurin on promoting the anti-tumor activity of anti-PD-1 against colon cancer.

## 2. Results

### 2.1. Midostaurin on Inhibited Mouse and Human Colorectal Carcinoma Cell Growth

**Figure 1:**
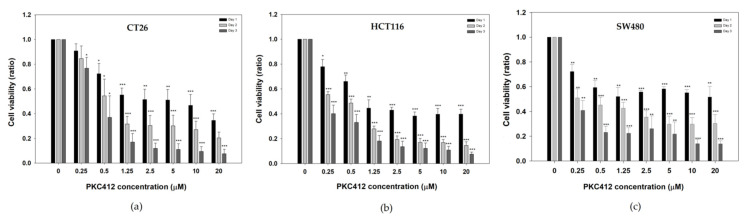
Cell viability of mouse and human colorectal adenocarcinoma after treatment with midostaurin. Mouse colorectal adenocarcinoma CT26 cells (**a**), human colorectal carcinoma HCT116 (**b**), and SW480 cells (**c**) were treated with different concentrations of midostaurin (PKC412) for 24, 48, and 72 h. Cell viability was measured by MTT reduction assay. Data from four separate experiments were expressed as mean ± standard error of the mean (SEM). Significant differences between control cells and cells treated with PKC412 are indicated by * *p* < 0.05, ** *p* < 0.01, and *** *p* < 0.001.

To evaluate the effect of midostaurin on mouse and human colorectal cancer cells, cells were exposed to various concentrations of midostaurin ranging from 0 to 20 μM for 24, 48, and 72 h. Midostaurin inhibited the growth of CT26, HCT116, and SW480 cells in a dose- and time-dependent manner with IC_50_ 5.6, 3.6, and 8.8 μM at 24 h, respectively (Figure 1).

### 2.2. Midostaurin on Induced G2/M Phase Arrest and Polyploid Formation in Colorectal Carcinoma Cells

**Figure 2:**
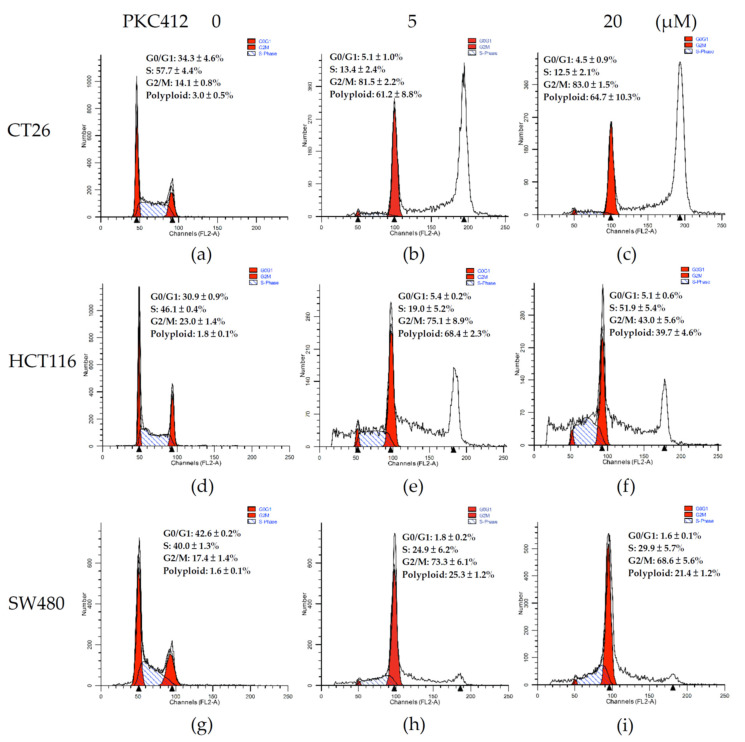
Cell-cycle analysis of colorectal adenocarcinoma cells treated with midostaurin (PKC412). Mouse and human colorectal adenocarcinoma cells were treated with midostaurin at 0 (**a**,**d**,**g**), 5 (**b**,**e**,**h**), and 20 μM (**c**,**f**,**i**) for 24 h. After treatment, cells were fixed and stained with propidium iodide. Cell-cycle distribution was acquisitioned by flow cytometry. Representative DNA histograms of colorectal cells treated with PKC412 were shown and the expression percentage of each cell-cycle phase was indicated in the panels. Data from four separate experiments were expressed as mean ± SEM.

By using DNA histogram analysis, the midostaurin profoundly affected the cell-cycle distribution of CT26 cells (Figure 2a–c). The cell cycle was arrested in the G2/M phase up to 83.0 ± 1.5% in midostaurin 20 μM treatment; different from 14.1 ± 0.8% in the control. The G2/M phase arrest was concomitant with the proportion of the hyperploid population formation up to 64.7 ± 10.3%; 3.0 ± 0.5% in the control group (Figure 2a,c). In human colorectal carcinoma cells, midostaurin also arrested cell cycle at the G2/M phase and induced polyploid formation in HCT116 and SW480 cells (Figure 2d–i). The proportion of the G2/M phase is 23.0 ± 1.4%, 17.4 ± 1.4% in the control group, and elevated to 37.7 ± 4.0% and 68.6 ± 5.6% in the midostaurin 20 μM treatment group in HCT116 and SW480 cells, respectively. The amount of polyploid population was increased from 1.8 ± 0.1% and 1.6 ± 0.1% in the control to 33.7 ± 4.0% and 21.4 ± 1.2% in the midostaurin-treated group in HCT116 and SW480 cells, respectively (Figure 2d,f,g,i). 

### 2.3. Midostaurin-Induced Micronuclei Formation in CT26 Cells

**Figure 3:**
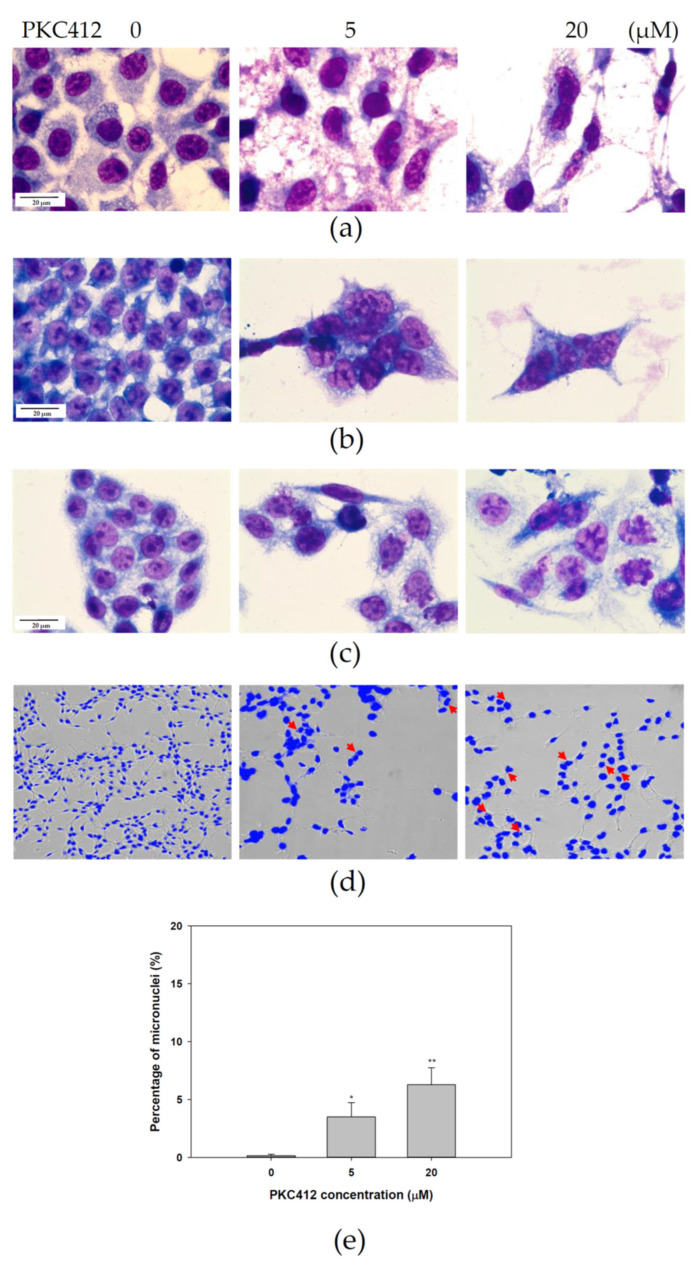
Morphology examination of colorectal carcinoma cells treated with midostaurin (PKC412). After treatment, morphologies were stained with Liu’s stain and photographed under light microscopy in 1000-fold magnification. The representative morphology of CT26 (**a**), HCT116 (**b**), and SW480 (**c**) cells was shown after staining. For micronucleic detection, CT26 cells were washed with PBS and fixed in 4% paraformaldehyde. The slides were subsequently stained with 1 μg/mL of DAPI for 10 min. The morphology and fluorescence expression were visualized and photographed under ImageXpress^®^ Micro 4 microscope in 200-fold magnification (**d**). Arrow (red) indicates the micronucleic formation. The proportion of micronucleic induced by PKC412 was counted (**e**). Data from three separate experiments were expressed as mean ± SEM. Significant differences between control cells and cells treated with PKC412 are indicated by * *p* < 0.05 and ** *p* < 0.01.

The morphology assessment by Liu’s staining showed the development of multinucleation in midostaurin-treated cells for 24 h in mouse and human colorectal adenocarcinoma cells (Figure 3a–c). The formation of micronuclei was also noted in midostaurin-treated cells, which was validated by DAPI immunofluorescence in CT26 cells (Figure 3d). The proportion of micronucleic cells induced by midostaurin in CT26 cells was increased from 0.14 ± 0.14% in the control group to 3.48 ± 1.24% and 6.29 ± 1.46% in the treatment group, with 5 and 20 μM of midostaurin, respectively (Figure 3e).

### 2.4. Midostaurin-Triggered Cytosolic DNA, DNA-Sensing Signaling, and Molecular Target Proteins Expression in Colorectal Adenocarcinoma Cells

**Figure 4:**
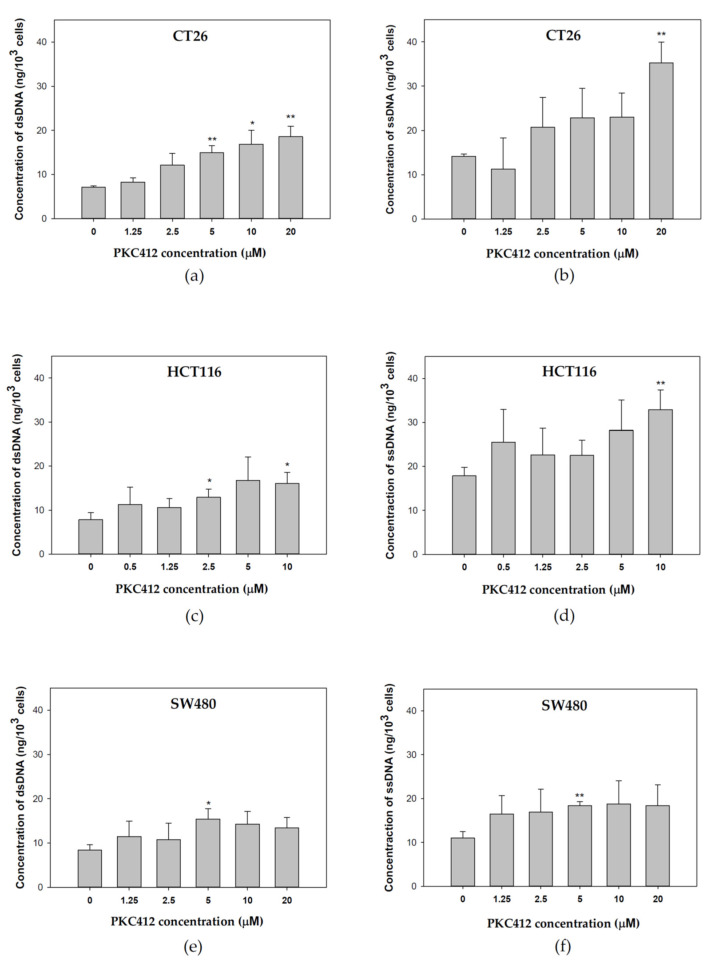
Cytosolic DNA expression profiles after midostaurin (PKC412) was administered in colorectal adenocarcinoma cells. CT26 (**a**,**b**), HCT116 (**c**,**d**), and SW480 (**e**,**f**) cells were grown in monolayer and treated with midostaurin for 24 h. After treatment, cells were detached, harvested, and the cell pellet was added with lysis solution to disrupt the cells. The samples were then centrifuged at 1200 rpm for 5 min and the supernatant containing cytosolic DNA was collected. The double-strand DNA (dsDNA) (**a**,**c**,**e**) and single-strand DNA (ssDNA) (**b**,**d**,**f**) were measured at 260 nm wavelength. Data from three separate experiments were expressed as mean ± SEM. Significant differences between control cells and cells treated with PKC412 are indicated by * *p* < 0.05 and ** *p* < 0.01.

**Figure 5:**
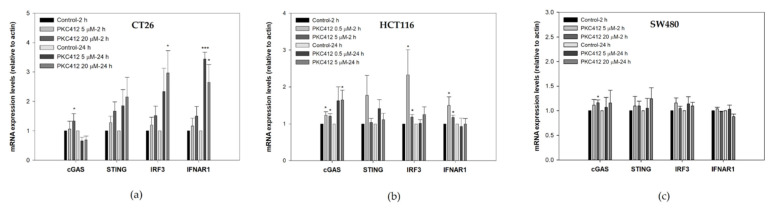
mRNA expression levels in CT26 (**a**), HCT116 (**b**), and SW480 (**c**) cells after treatment with midostaurin (PKC412). After treatment, RNA samples were isolated, followed by reverse transcription of RNA using primers of DNA-sensing specific genes. Data from four independent experiments were expressed as mean ± SEM. Significant differences between control cells and cells treated with PKC412 are indicated by * *p* < 0.05 and *** *p* < 0.001.

**Figure 6:**
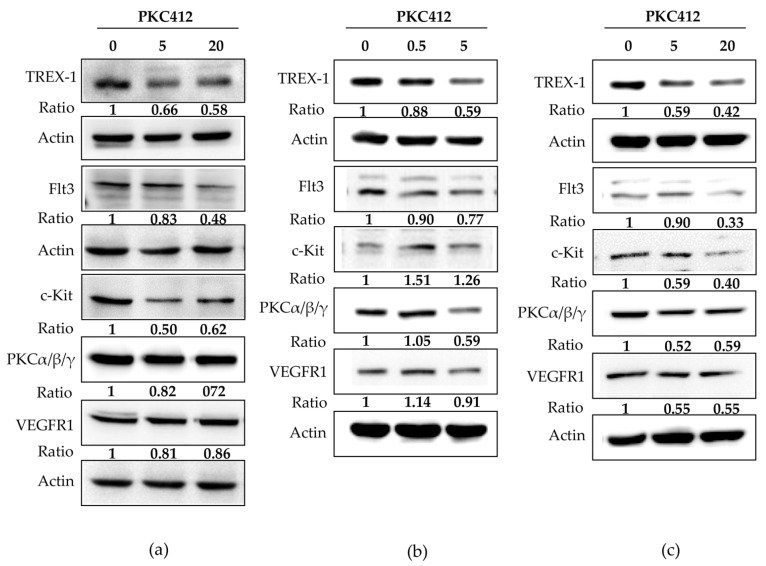
Effect of midostaurin (PKC412) on expression of putative target proteins in CT26 (**a**), HCT116 (**b**), and SW480 (**c**). Cells were treated with PKC412 for 24 h and then cells were lysed and harvested. Equal amounts of proteins were subjected to immunoblotting to detect midostaurin targets and Trex-1 proteins. Representative blots of midostaurin target proteins were shown. The densitometric analysis of each band was shown in the bottom panel. Original Western Blot images can be found in Figure S1.

In CT26 cells treated with midostaurin, the formation of cytosolic double-strand DNA was increased in a dose-dependent manner after treatment for 24 h (Figure 4a). However, midostaurin significantly induced single-strand DNA formation at a higher concentration of 20 μM midostaurin (Figure 4b). In human HCT 116 and SW480 colorectal adenocarcinoma cells, the levels of cytosolic double-strand DNA and single-strand DNA were also increased after various concentrations of midostaurin treatment. The midostaurin significantly induced double-strand and single-strand DNA formation at a higher concentration of 10 μM midostaurin in HCT cells but at a moderate concentration of 5 μM midostaurin in SW480 cells. In cytosolic DNA-sensing signaling, the mRNA expressions of the stimulator of interferon genes (STING), interferon regulatory factor 3 (IRF3), and interferon (alpha and beta) receptor 1 (IFNAR1) were up-regulated at 5 and 20 μM after midostaurin treatment for 24 h, whereas the cyclic GMP-AMP synthase (cGAS) was only up-regulated by 20 μM at 2 h in CT26 cells (Figure 5a). In HCT116 cells, cGAS, IRF3, and IFNAR1 mRNA expressions were up-regulated at 0.5 and 5 μM after midostaurin treatment for 2 h and only the cGAS mRNA expression was increased after midostaurin treatment for 24 h (Figure 5b). In SW480 cells, the expression of STING, IRF2, and IFNAR1 mRNA did not showed any changes after midostaurin treatment for 2 or 24 h whereas only cGAS mRNA was up-regulated after 20 μM midostaurin treatment for 2 h (Figure 5c). Trex-1 is a DNA exonuclease known to degrade cytosolic DNA. Midostaurin down-regulated the protein expression of Trex-1 in all three cells (Figure 6), which might be compatible with an increase in cytosolic DNA (Figure 4). For the molecular targets regarding midostaurin, the protein expressions of c-KIT and Flt3, but not PKCα/β/γ and VEGFR1, were down-regulated, indicating a possible off-target mechanism (Figure 6).

### 2.5. Midostaurin Abolished Trex-1 Protein Expression after FLT3L Activation

**Figure 7:**
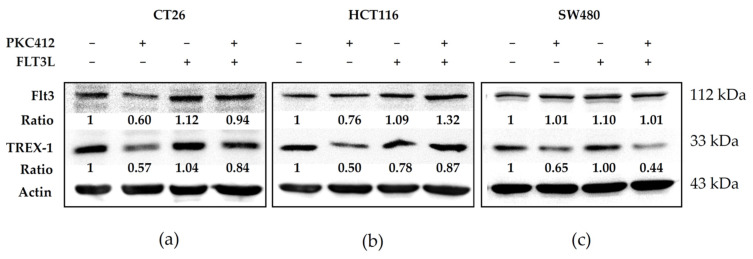
Effect of Trex-1 protein in midostaurin (PKC412)/FLT3L-treated colorectal adenocarcinoma cells. CT26 (**a**), HCT116 (**b**), and SW480 (**c**) cells were treated with PKC412 or/with FLT3L for 24 h and then cells were lysed and harvested. Equal amounts of proteins were subjected to immunoblotting to detect Flt3 and Trex-1 proteins. Representative blots of target proteins were shown. The densitometric analysis of each band was shown in the bottom panel. Original Western Blot images can be found in Figure S1.

Next, we investigated the role of the Trex-1 protein in the tyrosine inhibitor midostaurin-mediated cGAS-STING pathway. Midostaurin suppressed Flt3 and Trex-1 activation, and FLT3L activated both of these proteins in three colorectal adenocarcinoma cells. The Trex-1 protein was inhibited by a midostaurin plus FLT3L treatment (Figure 7). 

### 2.6. Midostaurin on Molecular Target Protein Expression in Spleen Macrophages

**Figure 8:**
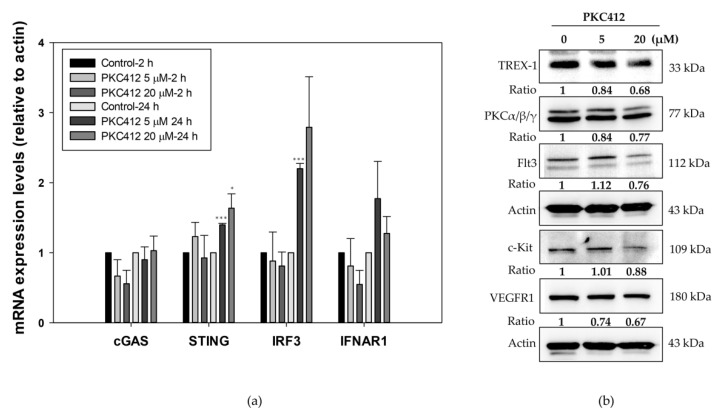
Effect of DNA-sensing signaling and molecular target proteins in midostaurin (PKC412)-treated spleen macrophages. Macrophages were treated with PKC412 for 2 or 24 h and then cells were lysed and harvested. RNA samples were isolated followed by reverse transcription of RNA using primers of DNA-sensing specific genes (**a**). Equal amounts of proteins were subjected to immunoblotting to detect midostaurin targets and Trex-1 proteins. Representative blots of midostaurin target proteins were shown (**b**). The densitometric analysis of each band was shown in the bottom panel. Data from three mice spleen macrophages were expressed as mean ± SEM. Significant differences between control cells and cells treated with PKC412 are indicated by * *p* < 0.05 and *** *p* < 0.001. Original Western Blot images can be found in Figure S1.

On the spleen macrophages as one lineage of antigen presenting cells, midostaurin augmented the mRNA expressions of STING, IRF3, and IFNAR1, but not cGAS, similar to that of the CT26 cells (Figure 8a). The protein expressions of Trex-1, c-KIT, Flt3, PKCα/β/γ, and VEGFR1 were similar to that in the CT26 cells (Figure 8b).

### 2.7. Combination of Midostaurin with Anti-PD-1 against Syngeneic CT26 Implanted Tumor In Vivo

**Figure 9:**
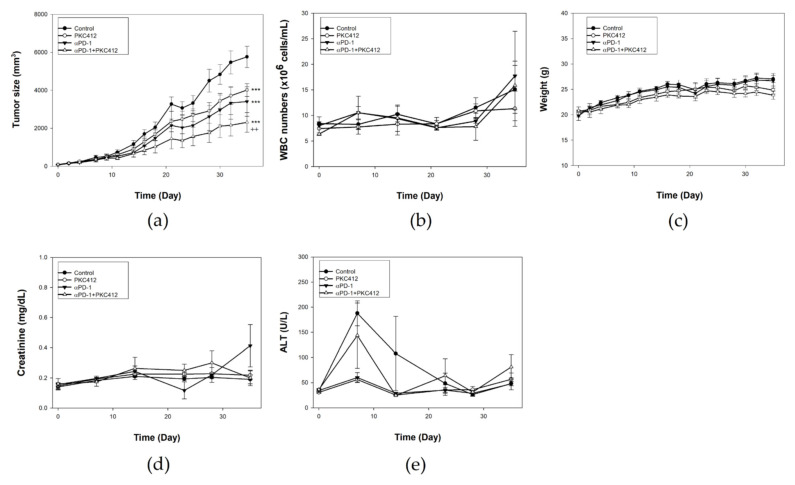
Therapeutic effect and toxicity in a CT26 syngeneic animal model treated with midostaurin (PKC412), anti-PD-1, and combination of midostaurin and anti-PD-1. BALB/c mice were implanted with CT26 colon cancer cells for tumor growth. After seven days, mice were administered with PKC412 100 mg/kg, anti-PD-1 antibody (200 μg), or a combination of PKC412 with anti-PD-1 antibody. Tumor volume was recorded using electronic caliper (**a**). Biological toxicities were examined by white blood cell counts (**b**), body weight (**c**), renal function with creatinine (**d**), and liver function with alanine transaminase (ALT) (**e**). Data from six mice of each group were expressed as mean ± SEM. Significant differences between the control group and the drug-treated group are indicated by *** *p* < 0.001. Significant differences between the monotherapy group and the combination group are indicated by ++ *p* < 0.01.

In vivo experiments revealed that both midostaurin (100 mg/Kg) and anti-PD-1 possessed moderate growth inhibition activity in an implanted CT26 tumor. The combination of midostaurin and anti-PD-1 had the greatest growth inhibition on the CT26-implanted tumor volume in comparison with each treatment alone (Figure 9a). Toxicity assessment by checking white blood cell count (Figure 9b), body weight (Figure 9c), creatinine (Figure 9d), and alanine transaminase (ALT) level (Figure 9e) showed no significant abnormality of midostaurin or combination.

### 2.8. Analysis of Immune Cell Profiles in Tumor Microenvironment and Spleen In Vivo

**Figure 10:**
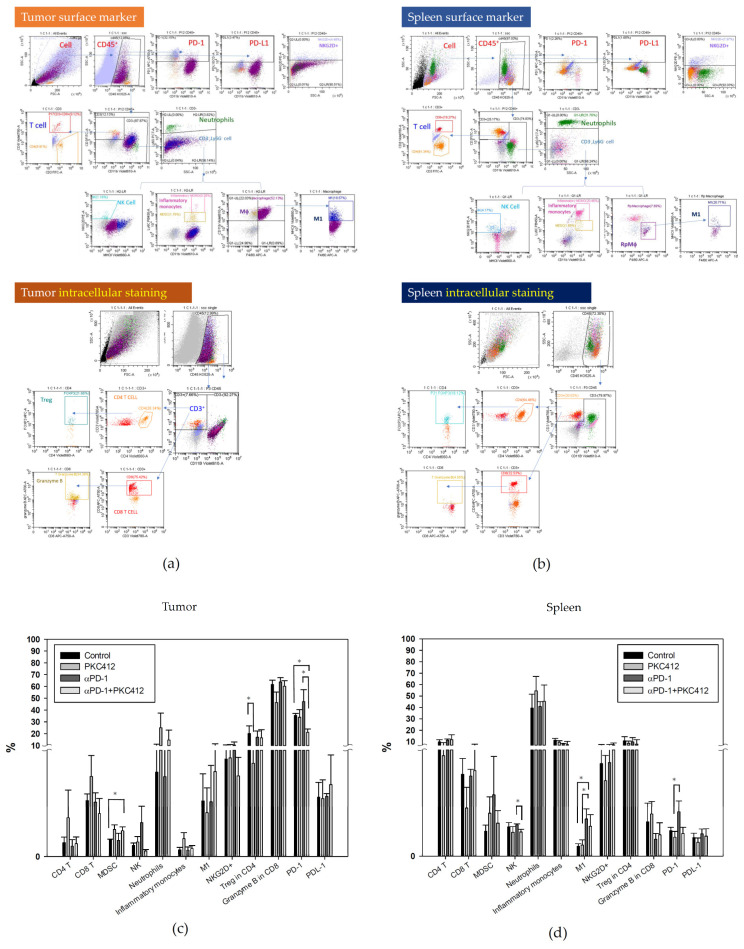
Expression profiles of circulating immune cells in a CT26 syngeneic animal model after monotherapy or combination of midostaurin (PKC412) and anti-PD-1. At the end of the experiment, mice were sacrificed and tumor and spleen specimens were harvested for flow cytometry analysis immune cell expression. The gating strategy of tumor and spleen specimens for flow cytometry analysis was shown (**a**,**b**). Quantification of immune cell profiles in tumor (**c**) and spleen (**d**) on day 39 after tumor cells implanted in syngeneic BALB/c mice. Data from six mice of each group were expressed as mean ± SEM. Significant differences between control group and PKC412-treated group, or anti-PD-1-treated group and combination-treated group are indicated by * *p* < 0.05.

In the immune cell profile analysis by multi-parameter flow cytometry, the cell lineages in a tumor for TME and a spleen for circulating cells were assessed. The gating strategy for flow cytometry analysis of the tumor and the spleen was indicated in Figure 10a,b. Midostaurin alone increased neutrophils and inflammatory monocytes and decreased Treg. It suppressed the anti-PD-1 antibody-triggered neutrophils, NKG2D^+^, and PD-1 and increased MDSC when combined with anti-PD-1 in TME (Figure 10c). In the spleen, M1 macrophage and PD-1 expression were elevated when midostaurin was combined with anti-PD-1 antibody treatment compared with the control, and decreased NK cell in combination therapy. There was no trend to increase or decrease in other types of immune cells in monotherapy or combination therapy (Figure 10d).

### 2.9. Analysis of STING and IFNβ Expression in Tumor Specimens

**Figure 11:**
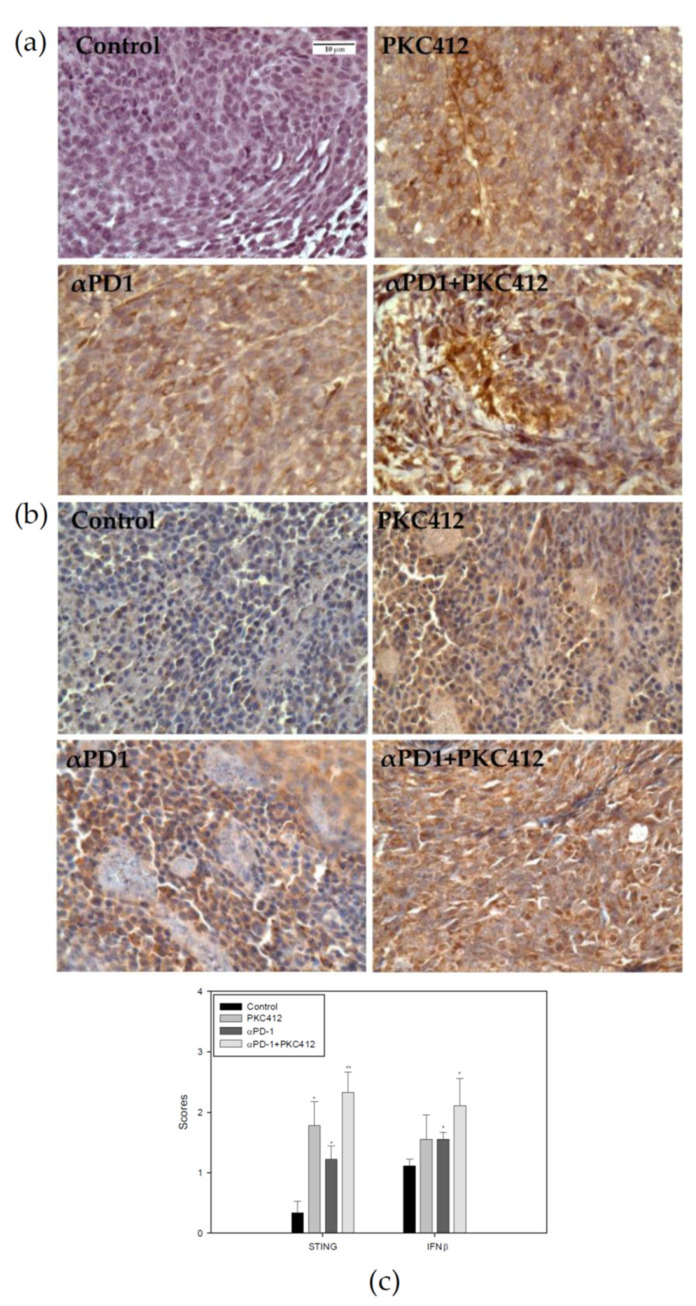
Expression levels of STING and IFNβ in a CT26 syngeneic animal model after monotherapy or combination of midostaurin (PKC412) and anti-PD-1. At the end of the experiment, mice were sacrificed and tumor specimens were harvested for immunohistochemistry staining STING (**a**) and IFNβ (**b**) protein expressions. The score of STING and IFNβ protein expressions level in tumor (**c**) after tumor cells implanted in syngeneic BALB/c mice. Data from six mice of each group were expressed as mean ± SEM. Significant differences between control group and PKC412-treated group, or anti-PD-1-treated group and combination-treated group are indicated by * *p* < 0.05, ** *p* < 0.01.

In the tumor specimens, STING and IFNβ protein expressions were analyzed using immunohistochemistry. Results showed that STING and IFNβ expressions were increased in the monotherapy and the combination therapy (Figure 11a,b). The extent of expression in the combination therapy is greater than the monotherapy (Figure 11c). 

## 3. Discussion

The novel effect of midostaurin on the enhancement of anti-PD-1 efficacy against colon cancer was noted. The formation of cytosolic DNA and the stimulation of DNA-sensing signaling with the modulation of the tumor microenvironment by midostaurin, but not its known target therapeutic effect, might be associated with the anti-PD-1-enhancing activity. 

Given that cytosolic DNA plays an important role in activating the cGAS-STING-type I IFN signaling in tumor cells, we utilized the production of cytosolic DNA as an index to screen candidate drugs for an enhancer of anit-PD-1. Midostaurin was selected for combination with anti-PD-1 due to the significant production of ssDNA and dsDNA in colorectal adenocarcinoma cells (Figure 4). The further evaluation for midostaurin on cGAS-STING-type I IFN signaling validated its effects on up-regulating the mRNA expression of cGAS, STING, IRF3, and IFNAR1 (Figure 5). This effect might be independent of its reported activities on regulating PKCα/β/γ and VEGFR1 (Figure 6). The genomic instability plays an important role in colon cancer, associated with metastasis, immune evasion, and therapeutic resistance. Based on different forms of instability, colorectal cancer can be categorized into two groups. One is a mismatch-repair deficiency with high-level microsatellite instability (MSI-H), the other is a mismatch repair proficient with low-level microsatellite instability (MSI-L) that is microsatellite-stable (MSS) [29,30]. In our model, mouse CT26 colorectal adenocarcinoma cell lines and human colorectal SW480 cells were categorized as MSS [31,32,33], while human colorectal adenocarcinoma cell HCT116 expressed MSI-H [34]. Chromosomal instability promotes metastasis by the formation of micronuclei, the response to cytosolic DNA, and finally constitutive activation of the cGAS–STING pathway in CT26 cells and the animal model [35]. In our results, we also found CT26 cells exhibited micronuclei formation, cytosolic DNA release, and cGAS-STING pathway activation in both the cellular and the animal models. However, another MSS cell line SW480 showed a lesser content of cGAS-STING activation. This may correlate to the STING signaling suppression impeding DNA damage responses [36]. The different DNA repair-system efficiency could be contributed to the discrepancy of the expression of the cGAS-STING pathway in these three cell lines. Other factors affecting the amount of cytosolic DNA such as direct diffusion mobility, exosome-mediated transportation, or enzymatic digestion may also cause the fluctuation of the cytosolic DNA level with different elevation patterns. [37,38,39]. The optimal condition of cytosolic DNA and cGAS-STING pathway protein expressions in colorectal cancer lines could be further clarified for clinical therapy. 

The drug repositioning involving the investigation of existing drugs for new therapeutic indications is an important and rational strategy in clinical pharmacology. In the present study, we found that midostaurin, a clinically available therapeutic agent against FLT3-mutated AML and KIT-mutated systemic mastocytosis, may be repositioned to an enhancer for anti-PD-1 against colon cancer. The advantages for drug repositioning include known established profile and management knowledge of adverse effects. However, the toxicity and the possible impact of midostaurin on immune-related adverse effect of anti-PD-1 remain to be investigated.

The down-regulation of Trex-1 expression by midostaurin was noted, along with an increase in the release of cytosolic DNA (Figure 6). These results may imply the DNA-damaging activity of midostaurin to produce DAMP and the simultaneous prevention from dsDNA degradation by Trex-1. This combinatory activity may amplify the effect of cytosolic DNA on activating cGAS-STING-type I IFN signaling to potentiate anti-tumor immunity.

In TME, midostaurin alone increased neutrophils and inflammatory monocytes and decreased Treg (Figure 10). In clinical practice, AML patients treated with midostaurin showed the reduction of Treg cells in peripheral blood mononuclear cells [40]. Our CT26 syngeneic animal model also showed Treg reduction after midostaurin treatment. This indicates that an FLT3 inhibitor midostaurin could affect T cell populations, especially Treg cells. NKG2D is a co-stimulatory receptor that defines a subpopulation of CD4^+^ T cells, producing high levels of proinflammatory IFN-γ and IL-17 upon stimulation [41]. Upon treatment by anti-PD-1, the secondary increase in neutrophils and MDSC known to suppress anti-tumor immunity was attenuated by midostaurin. Taken together, midostaurin may modulate an immune cell profile in TME towards a preferable one augmenting anti-PD-1 efficacy against cancer. The main effector cell lineages responsible for the anti-PD1-enhancing effect remains to be determined.

## 4. Materials and Methods 

### 4.1. Cell Culture

Mouse colon adenocarcinoma CT26 cells purchased from American Type Culture Collection (Manassa, VA, USA) and human colon adenocarcinoma HCT116 and SW480 cells were kindly provided by Prof. Ming-Jen Chen of MacKay Medical College. Cells were cultured in RPMI-1640 medium (GIBCO, Grand Island, NY, USA), with 10% fetal bovine serum (FBS) (Hyclone, Logan, UT, USA) and 2 mM of l-glutamine (Merck, Darmstadt, Germany) at 37 °C in a 5% CO_2_ incubator. Cells were kept in an exponential growth pattern. To prepare the mouse spleen macrophage, mice were euthanized and spleen was harvested. The spleen specimens were homogenized and passed through with 70 μm mesh sieve to obtain splenocytes. The cells were cultured in RPMI-1640 medium supplement with 20% FBS for growth.

### 4.2. FLT3L Activation

Cells were pre-treated with recombinant human Flt-3 ligand/FLT3L protein or recombinant mouse Flt-3 ligand/FLT3L protein (R&D Systems, Minneapolis, MN, USA) 100 ng for 24 h, then challenged with or without midostaurin 20 μM for 24 h. Cells were harvested and lysed for further Western blot analysis. 

### 4.3. MTT Assay

The MTT assay was used to estimate the cell viability. Briefly, cells were seeded in a 24-well plate and were treated with midostaurin for 24, 48, or 72 h. MTT was added into each well and incubated for 4 h at 37 °C. The medium was removed and 500 μL dimethyl sulfoxide (DMSO) was added into each well for 30 min to dissolve the formazan crystal. The absorbance of each well was measured (570/630 nm) and determined by an ELISA reader. All experiments were performed in triplicate and repeated three separated times.

### 4.4. Cell Cycle Analysis

Cells with different treatments were collected, washed with phosphate buffered saline (PBS), and fixed in cold 70% ethanol. Later, the cells were washed, re-suspended in cold PBS, and incubated with 10 mg/mL RNase and 1 mg/mL propidium iodide (PI) at 37 °C for 30 min in the dark. The samples were analyzed with flow cytometry (BD FACSCalibur, Becton Dickinson, Lincoln Park, NJ, USA). The percentage of the cancer cells in the G0/G1, S, G2/M, and polyploidy phases were estimated with Cell Quest software (Becton Dickinson). All experiments were triplicated.

### 4.5. Morphology Assessment

Cells were seeded on the 6-well plates and treated for 24 h. Then cells were washed with PBS twice and stained with the Liu’s stain solution A (Muto Pure Chemicals Co Ltd., Bunkyou-Ku, Tokyo, Japan) for 45 s followed by solution B for 90 s. Cell morphology was observed and photographed under BX51 light microscope (Olympus, Tokyo, Japan). For nuclear morphology, the cells were stained with 4′,6-diamidino-2-phenylindole (DAPI) and observed under ImageXpress Micro 4 microscope (Molecular Devices, San Jose, CA, USA).

### 4.6. Measurement of Cytosolic DNA

After treatment, cells were washed with PBS, trypsinized, and centrifuged to collect cells. The cell pellet was lysed with Chemicon^®^ Cytoplasmic Lysis Buffer (Merck Millipore, Burlington, MA, USA) for 15 min. The total DNA and single-strand DNA in cytoplasm were measured using NanoDrop2000C Spectrophotometer (Thermo Fisher Scientific Inc., Waltham, MA, USA) at 260 nm wavelength. The amount of double-strand DNA was calculated as the amount of total DNA minus the single-strand DNA.

### 4.7. Western Blot Analysis

Whole-cell lysates were prepared from cells treated with midostaurin using lysis buffer (Cell Signaling Technology, Danvers, MA, USA). Protein amounts were quantified using the bicinchoninic acid protein assay kit (Bio-Rad Laboratories, Hercules, CA, USA). Equal amount proteins were subjected with 10% sodium dodecyl sulfate-polyacrylamide gel electrophoresis (SDS-PAGE) and transferred to a polyvinylidene difluoride membrane. The blotting membrane was blocked with 5% defatted milk and then immunoblotted with primary antibodies including Flt3, c-Kit, PKCα/β/γ, VEGFR1 (Abcam, Cambridge, UK), Trex-1 (Cell Signaling Technology), and actin (GeneTex, Irvine, CA, USA) at 4 °C overnight. The membrane followed by the addition of horseradish peroxidase-labeled secondary antibodies (Jackson ImmunoResearch, West Grove, PA, USA) and developed using the enhanced chemiluminescence system (GeneTex). The results were analyzed using the Multigel-21 Multi-Function Gel Image System (Top Bio Co., Lin Kou, New Taipei City, Taiwan). The expression of β-actin was used as an internal control. The intensity ratio of immunoblot images were quantified using the ImageJ software.

### 4.8. mRNA Expression Assessment

Total RNA extraction was extracted from treated cells using RNAzol^®^ RT reagent (Molecular Research Center, Inc., Cincinnati, OH, USA). One microgram of RNA was used to synthesize cDNA with Thermo Scientific™ RevertAid First Strand cDNA Synthesis Kit (Thermo Fisher). Quantitative RT-PCR was performed using LightCycler^®^ 96 Real-Time PCR System (Roche, Penzberg, Upper Bavaria, Germany). PCR cycles were carried out at 65 °C for 5 min, 42 °C for 60 min, 70 °C for 5 min, and 40 cycles were carried out at 95 °C for 3 s and 72 °C for 3 min. Primers for specific genes were used as follows: IRF3, forward 5′-CGGAGGCTTAGCTGACAAAGA-3′ and reverse 5′-ATGCTCTAGCCAGGGGAGGA-3′; cGAS, forward 5′-TGAACATGTGAAGATTTCTGCTCC-3′ and reverse 5′-TGACTCAGCGGATTTCCTCG-3′; IFNAR1, forward 5′-TTTAATCCTGCCGTAGCCCC-3′ and reverse 5′-GCCAGCTCCTCCAGTTAGTG-3′; STING, forward 5′-ACTGCCGCCTCATTGTCTAC-3′ and reverse 5′-ATGGGGGCATTCATGGTA-3′; actin, forward 5′-GCCAACCGTGAAAAGATGAC-3′ and reverse 5′-GAGGCATACAGGGACAGCAC-3′. Gene expression was normalized to the actin expression levels from the same sample.

### 4.9. Syngeneic Tumor Implantation Model

Male BALB/c mice at five weeks old were purchased from the National Laboratory Animal Center of Taiwan (Taipei, Taiwan) and housed under 12-h light/dark cycle under a specific pathogen-free facility. All experimental protocols were conducted in accordance with the regulations and were approved by the Experimental Animal Committee of MacKay Memorial Hospital (Approval number: MMH-A-S-106-01). The 4 × 10^6^ CT26 cells in 100 μL PBS were subcutaneously inoculated on the right hindlimb of the mice. After the tumors grew for 7 days to achieve a mean diameter of approximate 5 mm, mice were randomly allocated into 4 groups as follows: (1) control, (2) midostaurin (100 mg/kg three times a week via oral gavage), (3) intraperitoneal anti-PD-1 injection (200 μg per intraperitoneal injection every other day for total 3 times, RMP1-14, Bio X cell, Lebanon, NH, USA), and (4) midostaurin in combination with anti-PD-1.

### 4.10. Evaluation of Tumor Volume and Toxicity

The volume of the implanted tumor and the body weight of each mouse were measured every other day by a single observer. Tumor volume was estimated using the formula, 1/2ab^2^, where a is the largest and b is the smallest diameter of the tumor measured by using electronic calipers. The blood samples were collected from the retro-orbital fossa and the white blood cells were counted using an automatic Coulter counter (HEMAVET HV950; Drew Scientific, Inc., Miamin Lakes, FL, USA). The plasma levels of alanine aminotransferase (ALT) and creatinine were measured using Fuji Dri Chem Slide (FUJIFILM Corporation Asaka Technology Development Center, Minamiashigara-shi, Kanagawa, Japan) and detected by Fujifilm DryChem NX-500 analyzer (FUJIFILM Corporation, Tokyo, Japan).

### 4.11. Flow Cytometry Analysis of the Immune Cells

After treatment, the mice were euthanized with ketamine (100 mg/kg) and xylazine (10 mg/kg) and sacrificed. The tumor and spleen specimens were excised, cut into 2–4 mm pieces, and digested with a solution containing collagenase A (1.5 mg/mL) and DNase I (0.4 mg/mL) at 37 °C for 30 min. Cells were sieved with a 70-μm cell strainer to dissociate and collect single-cell suspensions. Red blood cells were lysed with an ammonium-chloride-potassium solution (Invitrogen, Waltham, MA, USA). To reduce non-specific binding, the cells were suspended in the Fc receptor block (1 μg/1 × 10^6^ cells; BD Bioscience, San Diego, CA, USA) at 37 °C for 1 h before staining with the cell surface markers. Then, the cells were stained with antibodies conjugated with indicated fluorochromes as follows, anti-CD45-Brilliant Violet 510^TM^, anti-CD3-FITC, anti-CD11b-Brilliant Violet 605^TM^, anti-NKG2D-PE, anti-Ly6G-PE/Cyanine7, anti-MHCII-Brilliant Violet 650^TM^, anti-Ly6C-Brilliant Violet 421^TM^, anti-CD8-Brilliant Violet 785^TM^, anti-granzyme B-Alexa Fluor^®^ 700, anti-Foxp3-Alexa Fluor^®^ 647, anti-PD-1-APC/Cyanine7, anti-PD-L1-PE/Dazzle^TM^ 594, and anti-F4/80-APC (BioLegend, San Diego, CA, USA), for 20 min on ice. After washing, the cells were analyzed using cytoFLEX 13-color cytometry (Beckman Coulter, Brea, CA, USA) and quantified using CytExpert analysis software (Beckman Coulter, Brea, CA, USA). Immune profiles were defined as: NKG2D^+^ (CD45^+^/CD11b^−^/CD3^+^/NKG2D^+^), natural killer (NK) cells (CD45^+^/CD3^−^/Ly6G^−^/MHCII^−^/NKG2D^+^), neutrophils (CD45^+^/CD3^−^/Ly6G^+^), myeloid-derived suppressor cell (MDSC) (CD45^+^/CD3^−^/Ly6G^−^/CD11b^+^/Ly6C^+^), M1 macrophage (CD45^+^/CD3^−^/Ly6G^−^/CD11b^+^/F4/80^+^/MHCII^+^), inflammatory monocytes (CD45^+^/CD3^−^/Ly6G^−^/CD11b^+^/Ly6C^++^), granzyme B (CD45^+^/CD11b^−^/CD3^+^/CD8^+^/granzyme B^+^), regulatory T cells (Treg) (CD45^+^/CD11b^−^/CD3^+^/CD4^+^/FoxP3^+^), PD-1 (CD45^+^/PD-1^+^), and PD-L1 (CD45^+^/PD-L1^+^).

### 4.12. Immunohistochemistry Staining

Tumor specimens were deparaffinized with xylene and gradually rehydrated with ethanol. The slides had heat-inactivated antigen retrieval at 100 °C for 30 min in 10 mM sodium citrate buffer (0.05% Tween20, pH 6.0) buffer. The samples were blocked using BlockPRO blocking buffer (Visual Protein, Taipei, Taiwan) and then incubated with primary antibodies against IFNβ (1:100, Abcam, Cambridge, UK) and STING (1:100, Cell Signaling Technology, MA, USA) at room temperature for 2 h. A negative control was also processed without primary antibodies. Endogenous peroxidase activity was blocked by 3% hydrogen peroxide at room temperature for 10 min. For enzymatic detection, SignalStain^®^ Boost IHC Detection Reagent (HRP, Rabbit) (Cell Signaling Technology) was used as a secondary antibody at room temperature for 30 min and developed with 3,3-diaminobenzidine (DAB) as chromogen for 10 min (Dako Liquid DAB + Substrate Chromogen System, Dako, CA, USA). The slides were counterstained with hematoxylin (Merck, Darmstadt, Germany), dehydrated, and mounted. Cells expressing STING and IFNβ proteins were visualized, and images were obtained from five different areas to assess the scores by microscope. The scores, using a 0–4 scale, were defined as follows: 0, no expression; 1, mild expression; 2, moderate expression; 3, severe expression.

### 4.13. Statistical Analysis

The data are expressed as mean ± standard error of the mean. Statistical comparison in the experiment was performed using one-way analysis of variance (ANOVA) followed by an LSD test for post hoc analysis and a Kruskal–Wallis test (K-W test). Additionally, for within-subject dependency owing to repeated measurements as the experiment went on, repeated ANOVA and a generalized estimating equation (GEE) method of multiple linear regression model was utilized to compare the between-group variables as statistically appropriate. All statistical analyses were performed using the SigmaPlot version 10.0 (Systat Software, Inc., San Jose, CA, USA). Significant differences between groups are indicated as * *p* < 0.05, ** *p* < 0.01, and *** *p* < 0.001.

## 5. Conclusions

In conclusion, midostaurin may have the potential to enhance anti-PD-1 efficacy against colon cancer accompanied by a modulation of the tumor microenvironment. 

## Data Availability

Data are contained within the article.

## Supplementary Materials

The following are available online at https://www.mdpi.com/article/10.3390/cancers14194847/s1, Figure S1: The original blot showing with molecular weight markers of western blot Figure 6 Figure 7 Figure 8 presented in the paper.
