# Supplementary material for: Midostaurin Modulates Tumor Microenvironment and Enhances Efficacy of Anti-PD-1 against Colon Cancer
Source: Cancers (Basel). 2022 Oct 4;14(19):4847. doi: 10.3390/cancers14194847 (PMC9563721; doi:10.3390/cancers14194847)

Supplementary Materials: Midostaurin Modulates Tumor Microenvironment and Enhances Efficacy of Anti-PD-1 against Colon Cancer

Cheng-Ta Lai, Chih-Wen Chi, Shu-Hua Wu, Hui-Ru Shieh, Jiin-Cherng Yen* and Yu-Jen Chen*

Figure S1. The original blot showing with molecular weight markers of western blot figures 6 to 8 presented in the paper.

Figure 6.

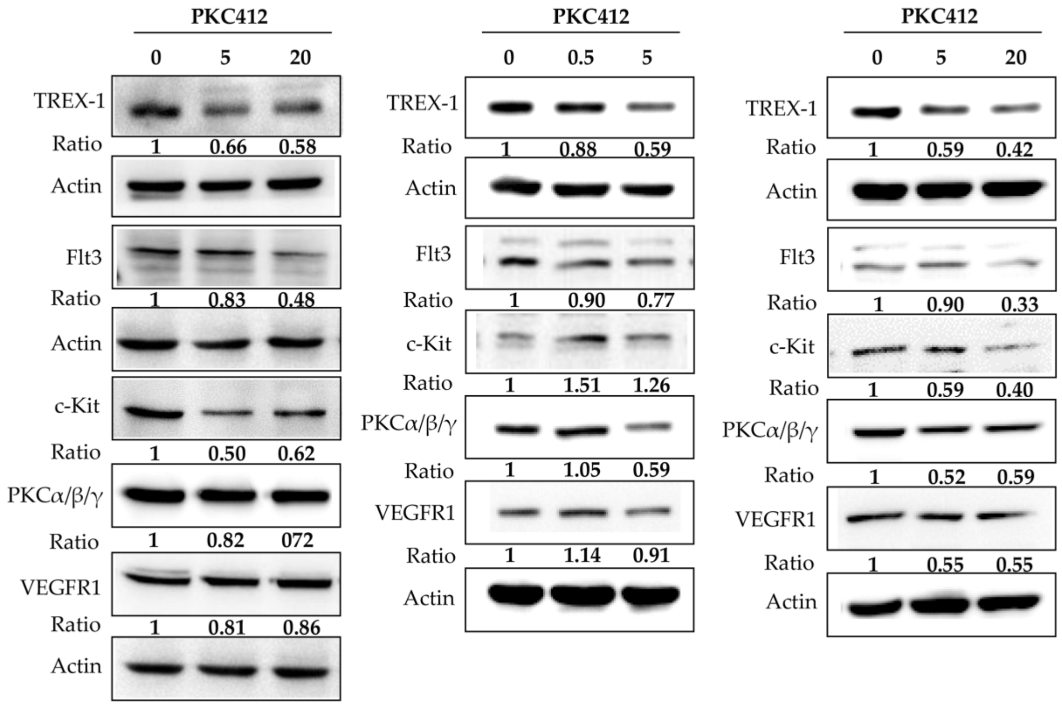

(a) (b) (c)

Original blots

CT26 cell

Trex-1 (33 kDa)

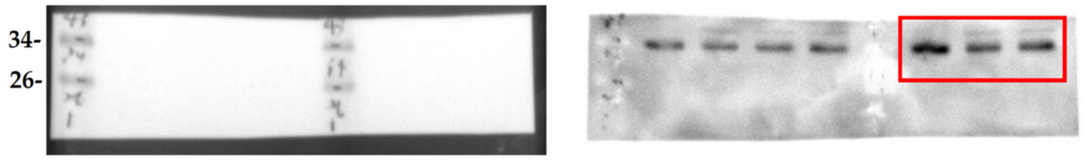

Actin (43 kDa)

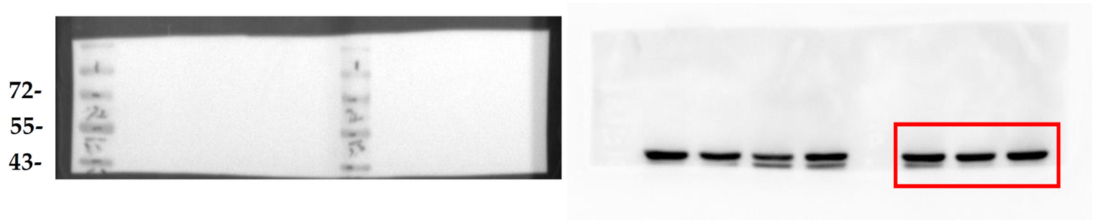

Flt3 (112kDa)

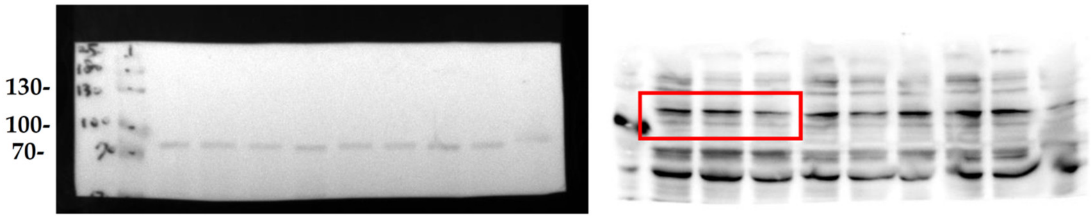

Actin (43 kDa)

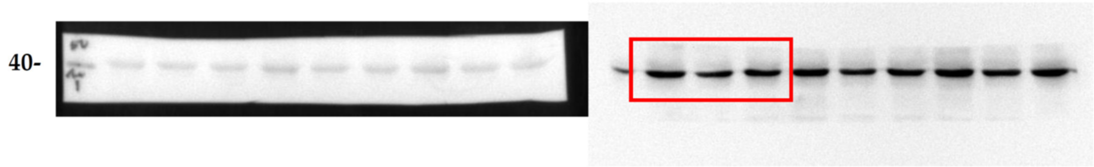

c-Kit (109 kDa)

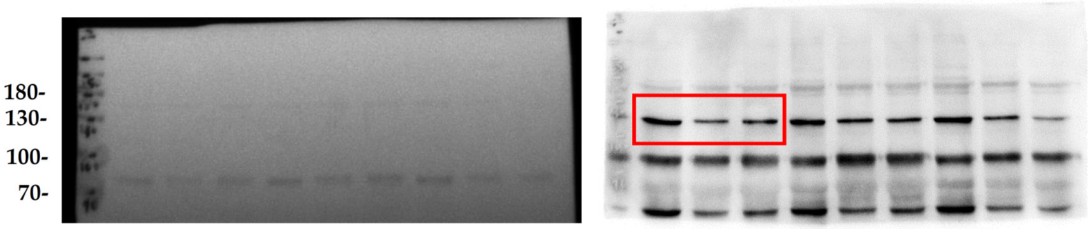

PKCα/β/γ (77 kDa)

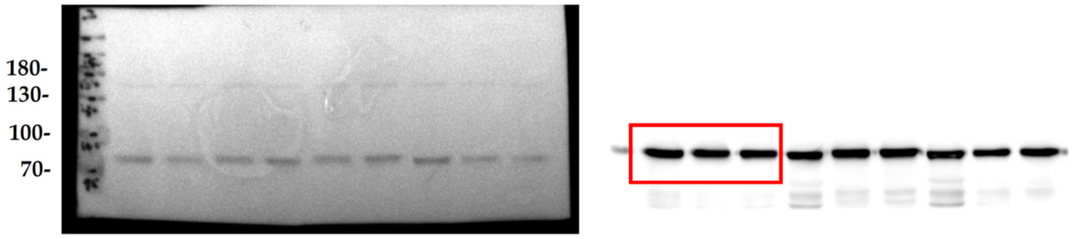

VEGFR1 (180 kDa)

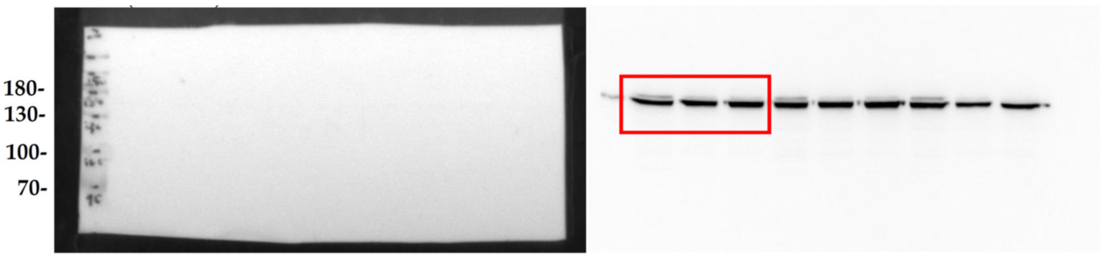

Actin (43 kDa)

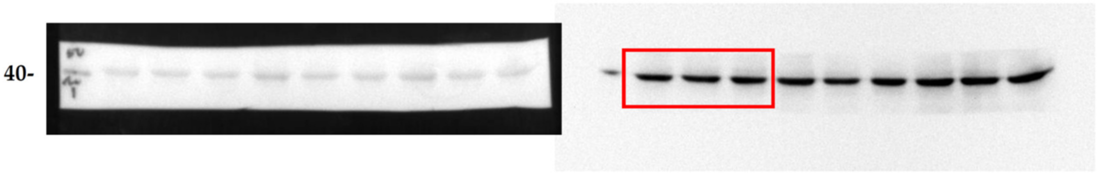

HCT116 cell

Trex-1 (33 kDa)

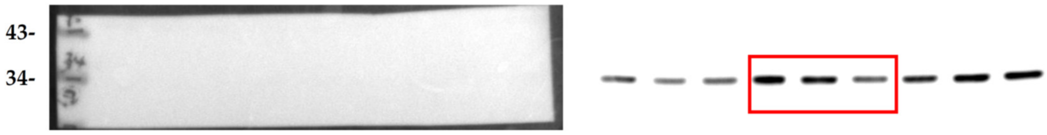

Actin (43 kDa)

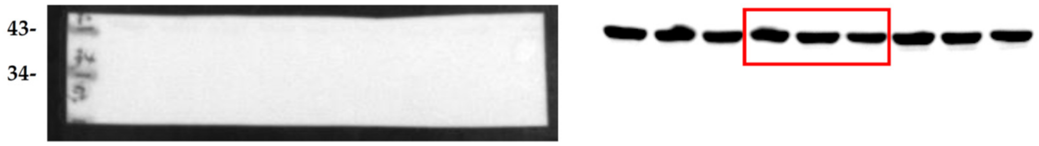

Flt3 (112 kDa)

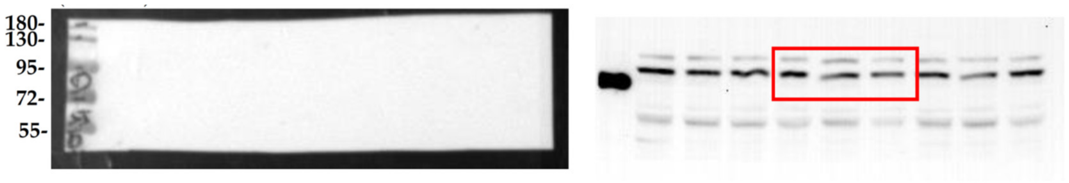

c-Kit (109 kDa)

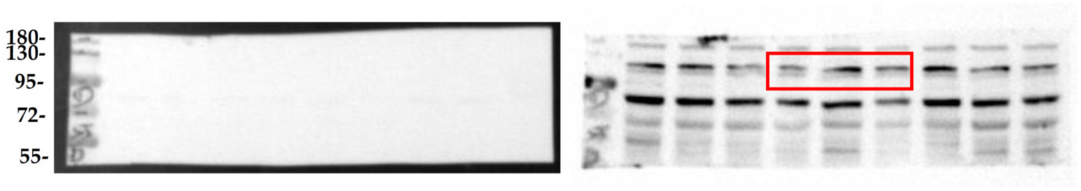

PKCα/β/γ (77 kDa)

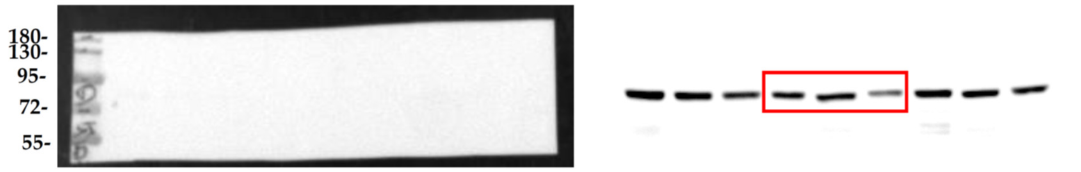

VEGFR1 (180 kDa)

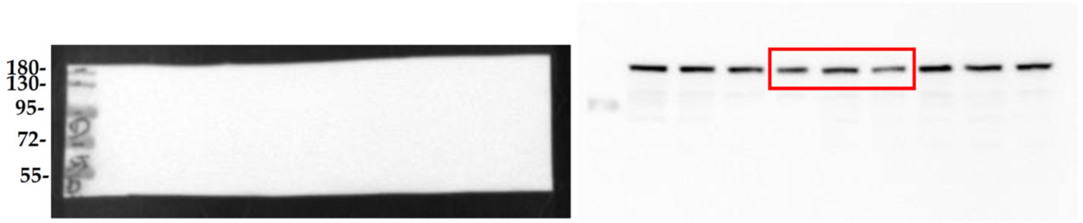

Actin (43 kDa)

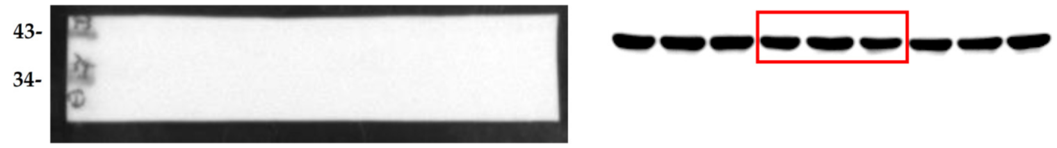

SW480 cell

Trex-1 (33 kDa)

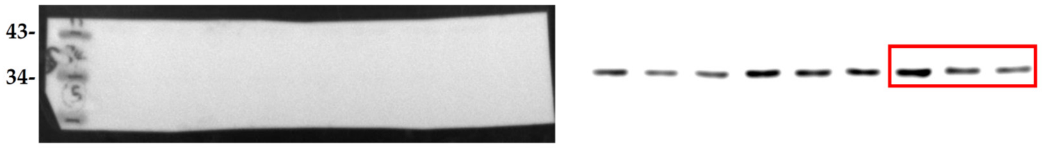

Actin (43 kDa)

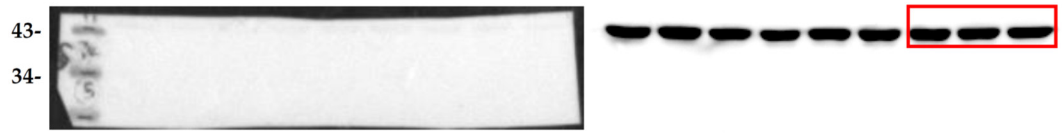

Flt3 (112 kDa)

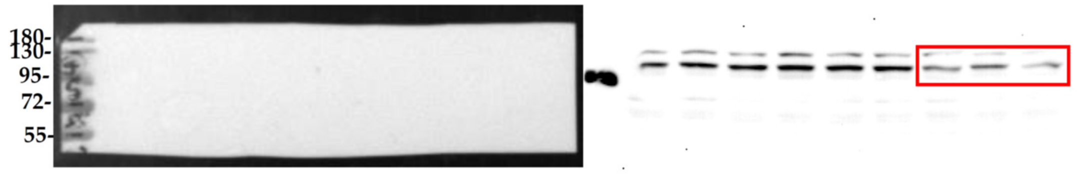

c-Kit (109 kDa)

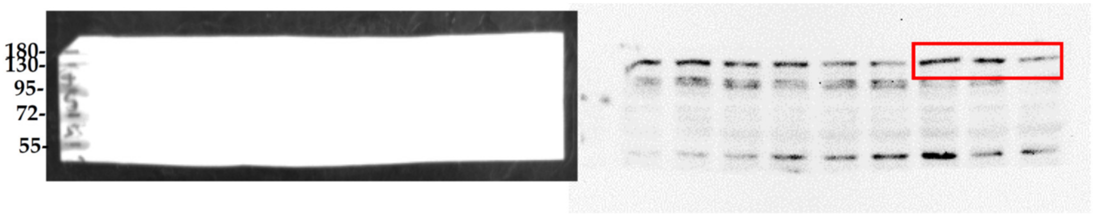

PKCα/β/γ (77 kDa)

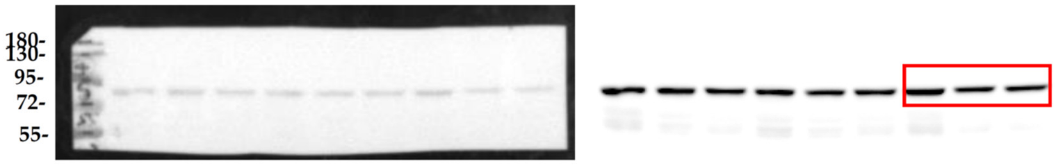

VEGFR1 (180 kDa)

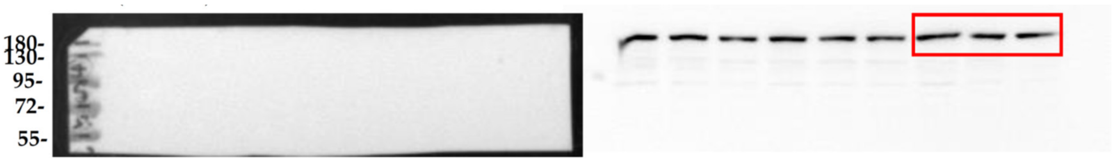

Actin (43 kDa)

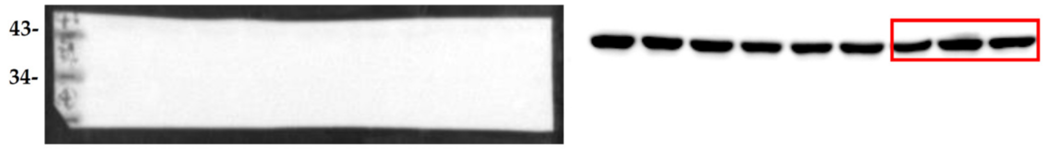

Figure 7

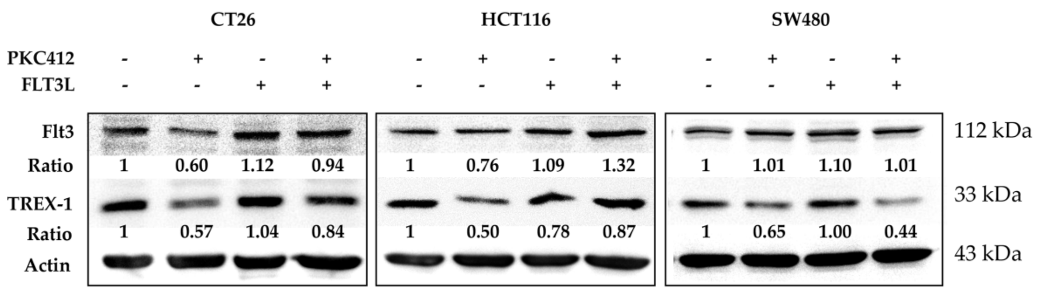

Original blots

CT26 cell

Flt3 (112 kDa)

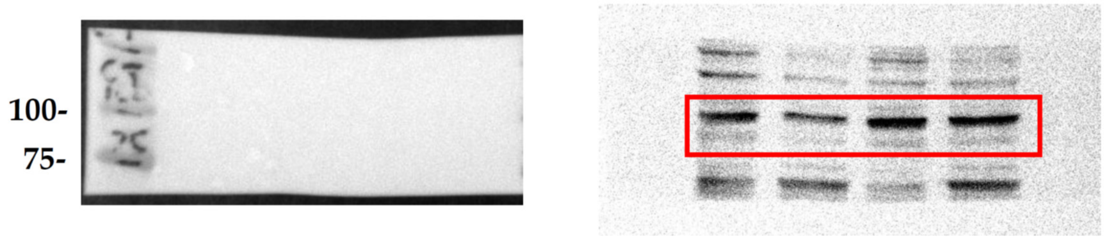

Trex-1 (33 kDa)

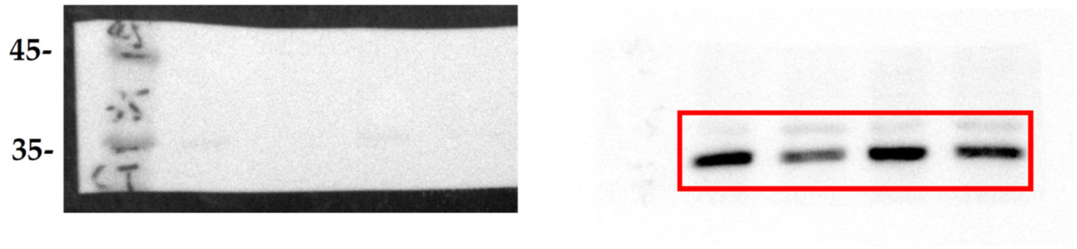

Actin (43 kDa)

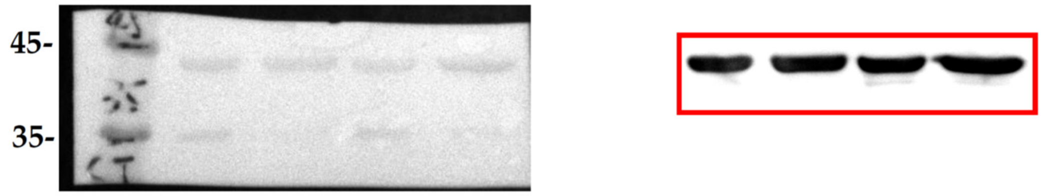

HCT116 cell

Flt3 (112 kDa)

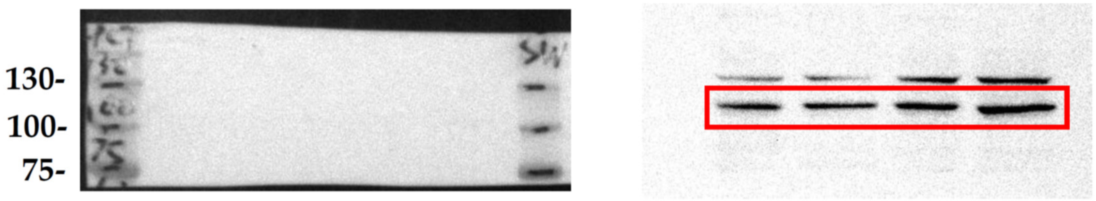

Trex-1 (33 kDa)

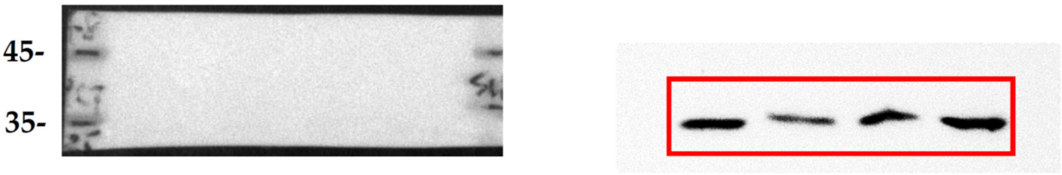

Actin (43 kDa)

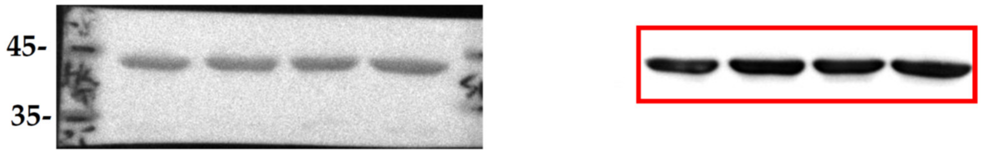

SW480 cell

Flt3 (112 kDa)

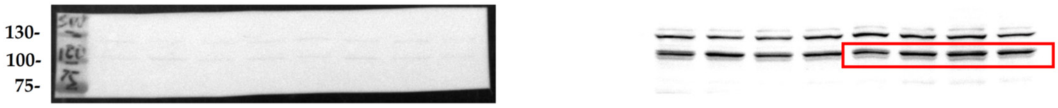

Trex-1 (33 kDa)

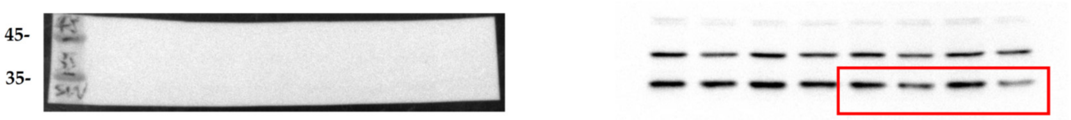

Actin (43 kDa)

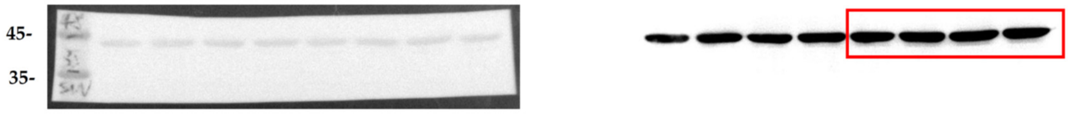

Figure 8.

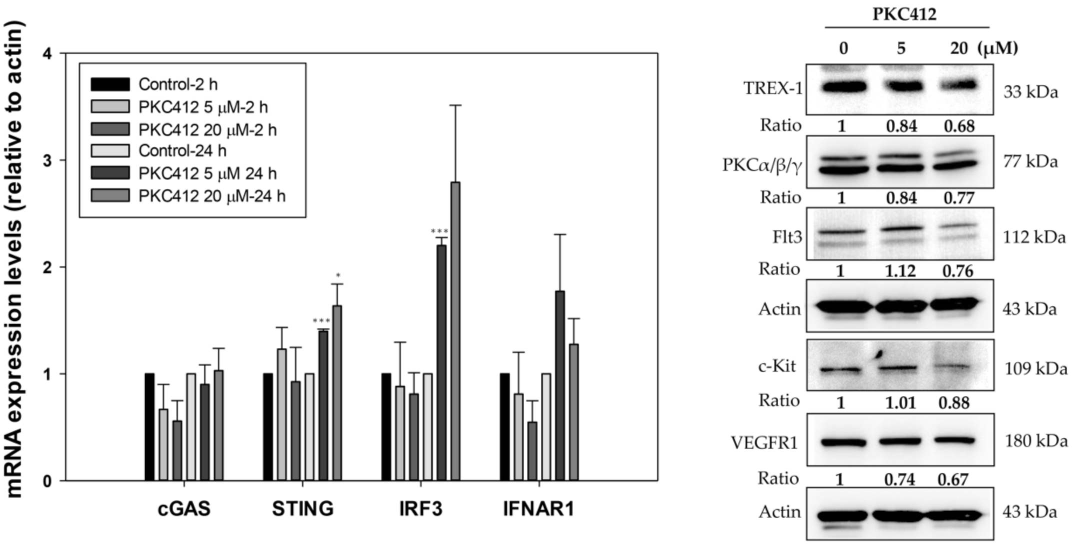

(a) (b)

Original blots

Trex-1 (33 kDa)

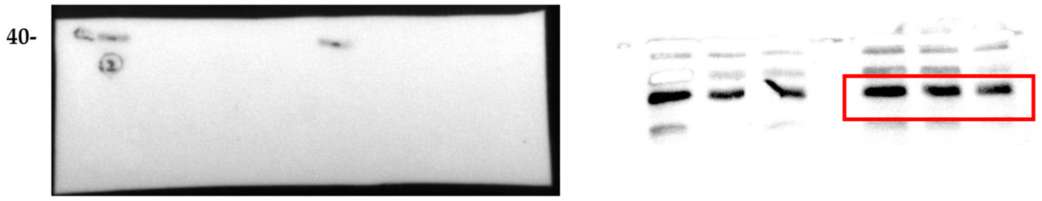

PKCα/β/γ (77 kDa)

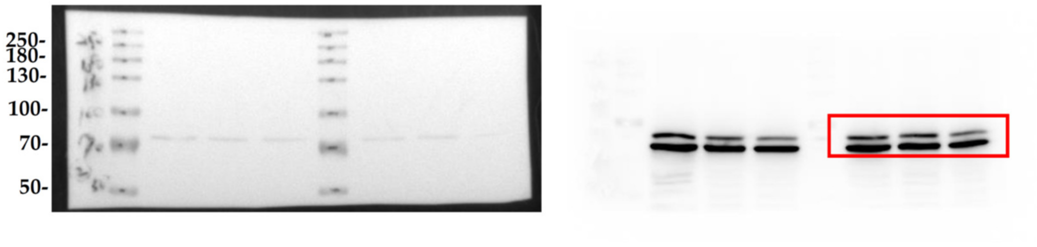

Flt3 (112 kDa)

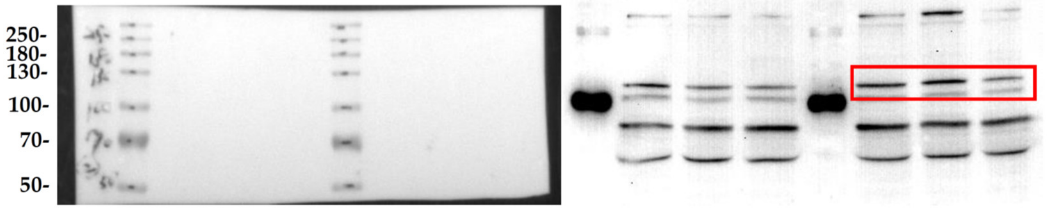

Actin (43 kDa)

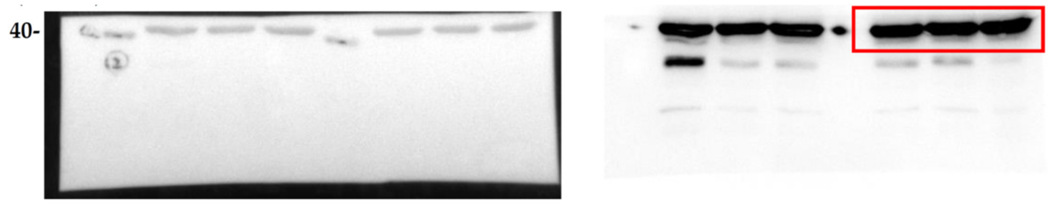

c-Kit (109 kDa)

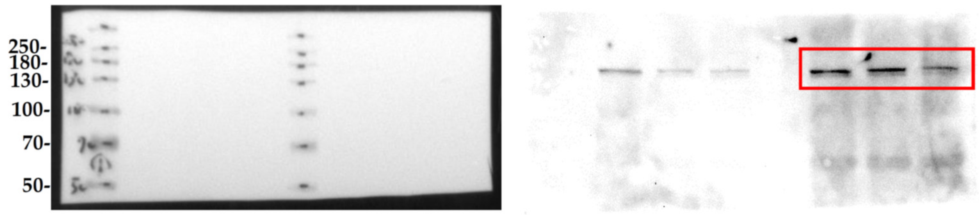

VEGFR1 (180 kDa)

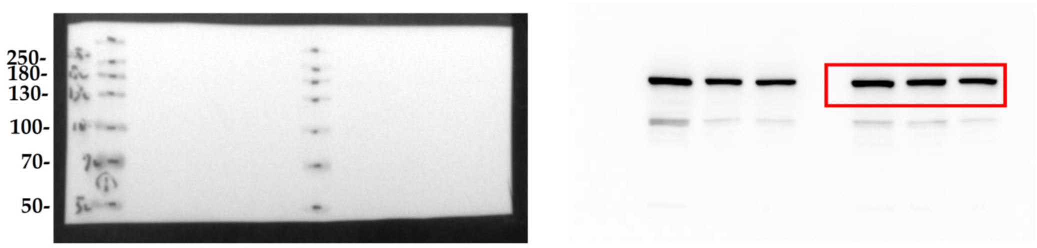

Actin (43 kDa)

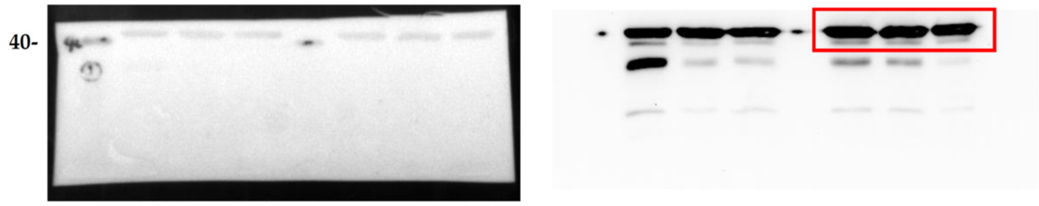

Supplement: Supplementary file 1 [file cancers-14-04847-s001.zip › cancers-1933691-supplementary.pdf]
